# Toward Molecular Stratification and Precision Medicine in Systemic Sclerosis

**DOI:** 10.3389/fmed.2022.911977

**Published:** 2022-06-30

**Authors:** Maria Noviani, Vasuki Ranjani Chellamuthu, Salvatore Albani, Andrea Hsiu Ling Low

**Affiliations:** ^1^Department of Rheumatology and Immunology, Singapore General Hospital, Singapore, Singapore; ^2^Duke–National University of Singapore Medical School, Singapore, Singapore; ^3^Translational Immunology Institute, SingHealth Duke-NUS Academic Medical Centre, Singapore, Singapore

**Keywords:** systemic sclerosis, stratification, precision medicine, molecular, multi-omic analyses

## Abstract

Systemic sclerosis (SSc), a complex multi-systemic disease characterized by immune dysregulation, vasculopathy and fibrosis, is associated with high mortality. Its pathogenesis is only partially understood. The heterogenous pathological processes that define SSc and its stages present a challenge to targeting appropriate treatment, with differing treatment outcomes of SSc patients despite similar initial clinical presentations. Timing of the appropriate treatments targeted at the underlying disease process is critical. For example, immunomodulatory treatments may be used for patients in a predominantly inflammatory phase, anti-fibrotic treatments for those in the fibrotic phase, or combination therapies for those in the fibro-inflammatory phase. In advancing personalized care through precision medicine, groups of patients with similar disease characteristics and shared pathological processes may be identified through molecular stratification. This would improve current clinical sub-setting systems and guide personalization of therapies. In this review, we will provide updates in SSc clinical and molecular stratification in relation to patient outcomes and treatment responses. Promises of molecular stratification through advances in high-dimensional tools, including omic-based stratification (transcriptomics, genomics, epigenomics, proteomics, cytomics, microbiomics) and machine learning will be discussed. Innovative and more granular stratification systems that integrate molecular characteristics to clinical phenotypes would potentially improve therapeutic approaches through personalized medicine and lead to better patient outcomes.

## Introduction

Systemic sclerosis (SSc) is a multi-system immune-mediated disease characterized by vasculopathy and fibrosis of skin and internal organs (1). Early clinical manifestations include Raynaud's phenomenon, puffy swollen fingers and gastroesophageal reflux (2, 3). Later manifestations include musculoskeletal involvement, severe vasculopathy such as digital ulcerations, gastrointestinal (GI) complications, interstitial lung disease (ILD), pulmonary arterial hypertension (PAH) and scleroderma renal crisis (4). Although uncommon, SSc has one of the highest morbidity and mortality among autoimmune diseases, with cumulative 10-year survival of 62% from diagnosis (1, 5). Unmet needs in the management of SSc include risk stratification to prognosticate severity of disease complications and to predict treatment responses.

The heterogenous pathological processes that define SSc is a challenge to targeting appropriate treatment. SSc subset classification into relatively homogenous subtypes would have prognostic value to stratify patients for disease complications and treatment responses. The classification of SSc subsets have relied mainly on clinical features. Incorporation of laboratory (e.g., autoantibodies) and molecular gene signatures could lead to a more granular classification system. This is a promising approach toward precision medicine.

The most commonly used SSc classification system is based on the extent of skin involvement, specifically limited cutaneous (lc) and diffuse cutaneous (dc) SSc (6). A minority (<5%) of patients have clinical features of SSc and SSc-specific antibody without any skin involvement, and this group is classified as sine-scleroderma (7). This SSc classification system is suitable for clinical care as it is mainly based on clinical examination of skin with fairly distinct clinical associations and specific serum auto-antibody profiles (8). Nevertheless, due to the heterogenous nature of SSc, patients may present with similar initial clinical manifestations, but have different clinical outcomes and responses to treatment. Thus, SSc classification system needs to be refined to incorporate laboratory tools, such as auto-antibody profiles and gene expression signatures.

Molecular stratification allows segregation of different groups of patients based on pathogenetically homogenous subsets in relation to organ manifestations, prognosis and treatment response. The pathogenesis of SSc involves a complex interplay between immune activation and vascular damage, which leads to activation of fibroblast and excessive collagen deposition in the skin and internal organ (9). Better understanding of variable contributions from each process during the course of the disease could help tailor treatment approaches for different patients.

Therapies used for different clinical manifestations of SSc were shown to have varied efficacy (10). Evidence-based treatment guidelines published in 2017 by EUSTAR adopt an organ-based approach, rather than one based on the patients' clinical, laboratory or molecular subsets (11). The diverse natural course of SSc disease makes it challenging to predict which patients will benefit the most from particular treatment based on clinical manifestations alone. Molecular stratification of SSc will help to personalize treatment based on distinct molecular signatures.

In this review article, we will provide updates in SSc clinical and molecular stratification in relation to patient outcomes and treatment responses. We will also discuss promises of molecular stratification through advances in high-dimensional tools, including deep phenotyping of tissues to single cell analysis, network medicine, omic-based stratification (transcriptomics, genomics, epigenomics, proteomics, cytomics, and microbiomics) and machine learning.

## SSc Classification System

SSc classification system is a rapidly evolving field. Over the years, a combination of multi-system involvement, SSc-specific autoantibodies, and nail-fold capillaroscopy (NFC) patterns have emerged to supplement SSc classification.

### SSc Classification Based on Cutaneous Involvement

The most commonly used system of SSc classification is a two-subset criteria by LeRoy et al., which dichotomizes patients into lcSSc and dcSSc based on extent and pattern of skin fibrosis (12). LcSSc, which includes patients with cutaneous involvement distal to the elbows or knees, is usually associated with anti-centromere antibody (ACA), telangiectasia and late onset of PAH (12). Whereas, dcSSc, which includes patients with cutaneous involvement proximal to the elbows or knees, is frequently associated with anti-topoisomerase I antibody (ATA), tendon friction rub, early internal organ involvement such as ILD, myocardial and diffuse GI tract involvement; hence, dcSSc is known to have poorer prognosis than lcSSc (12).

Although this classification system has a discriminatory value in the prognostication of patients, it has various limitations. There may be overlapping clinical features between the two subsets, e.g., ILD occurrence was 30% in lcSSc and 50% in dcSSc (*p* = 0.16) (13). A subgroup of patients may have serological, vascular and internal organ manifestations of SSc but without cutaneous involvement, and this subgroup has been classified as sine scleroderma (14). In addition, patients with very early diagnosis of systemic sclerosis (VEDOSS) may not have cutaneous involvement or internal organ involvement but have early SSc features such as Raynaud's phenomenon with vascular changes on NFC or SSc-specific autoantibody (2). A subset of patients may also display overlap syndromes with other connective tissue diseases (e.g., systemic lupus erythematosus and polymyositis), and be variably associated with anti-Ku, PM-Scl75 or anti-U1-ribonucleoprotein antibodies (15, 16).

### SSc Classification System Based on More Novel Disease Attributes

Combining clinical data with laboratory tools may provide better prognostic value and be feasibly applied in routine clinical care. NFC patterns have been demonstrated to have prognostic value to inform disease activity and disease progression. The abnormal NFC patterns are classified as early, active and late (17, 18). In an international multi-center cohort study evaluating cross-sectional data in the EUSTAR registry, early/active NFC patterns were found in patients with mild/moderate skin involvement and low number of disease manifestations; whereas late NFC pattern was associated with more severe forms of SSc disease (17, 18). Moreover, the NFC pattern could also be an indicator of overall disease progression (18). Prospective cohort study of SSc patients (*n* = 140) over 3 years showed that reduced capillary density was associated with overall disease progression, progression of skin fibrosis, occurrence of new digital ulcers and new onset PAH (18). Furthermore, the severity of NFC patterns was shown to be predictive of future severe organ involvement with increasing risk from early to late pattern, after adjusting for disease duration, subset and vasoactive medications (19, 20).

SSc-specific autoantibodies are strong predictors of disease outcome and internal organ involvement (21). The 3 main SSc-specific autoantibodies are ACA, ATA and anti-RNA polymerase III antibody (anti-RNAP III), and they are usually mutually exclusive (21, 22). SSc patients with ACA have better prognosis and are more likely to have limited cutaneous involvement and PAH (21). Patients with ATA represent a distinct subgroup with extensive cutaneous involvement and increased risk of ILD (21), and anti-RNAP III represents a subgroup with higher risk of malignancies and development of scleroderma renal crisis (21, 23). Other SSc-specific autoantibodies identified to have prognostic values include anti-Th/To, which is associated with lcSSc and ILD but lower prevalence of PAH (21, 24).

Integration of autoantibody profiles with clinical phenotypes can provide a more robust and comprehensive classification system to risk stratify the patients better. Using cluster analysis of a combination of auto-antibodies and clinical features in 140 SSc patients, Boonstra et al. revealed 5 subgroups of patients with different prognosis and clinical outcomes (25). However, autoantibodies only partially contributed to risk stratification as not all ATA-positive patients had worse prognosis. Another cluster analysis of a large database using clinical and serologic variables (120 EUSTAR centers, *n* = 6927) showed that dichotomous classification of SSc patients were insufficient as significant proportion of patients with lcSSc (39%) and dcSSc (19%) clustered discordantly. By using data on the presence of organ damage to prognosticate risk of more organ damage/ death, the study identified 6 different clusters with more homogenous clinical phenotypes (Table 1) (26). Although cluster analyses have improved current classification system for better risk stratification, the analysis was driven by data readily available to physicians and none of the analyses included high-throughput molecular data (26). Moreover, the mean disease duration of patients in the EUSTAR study was 11 years (26). We believe that incorporation of high throughput molecular data could improve SSc stratification system.

**Table 1:** Stratification in relation to clinical features.

	**Subset and association with clinical features**	**References**
Cutaneous	**Extent of skin involvement**	(12)
	lcSSc: higher prevalence of PAH and ACA	
	dcSSc: higher prevalence of ILD, less prevalence of ACA	
	**Pre-fibrotic stage /very early disease**	
	VEDOSS: (RP, puffy finger, ANA) AND (NFC or SSc-specific Ab)	(2)
NFC	Early/ active: mild/moderate skin involvement, low number of disease manifestations	(17)
	Late pattern: more severe disease	
	Reduced number of capillaries: overall disease progression, DU, PAH, ILD	(18–20)
SSc-Ab	ACA: lcSSc, PAH	(21)
	ATA: dcSSc, ILD	
	anti-RNAP III: lcSSc, SRC	
	anti-Th/ To: lcSSc, ILD, PAH	
	anti-U3RNP: dcSSc, muscle involvement, PAH	
	anti-PM-Scl: PM/DM overlap, arthritis overlap, ILD	
	anti-Ku: muscle and joint involvement	
	anti-U1RNP: overlap syndromes	
	anti-U11/ U12RNP: ILD	
Clinical features and prognosis	Cluster 1: female, older onset, GI involvement, lcSSc, ACA	(26)
	Cluster 2: ILD, PH, lcSSc, ACA, ATA	
	Cluster 3: younger onset, lowest mRSS, less aggressive, lcSSc, ACA > ATA	
	Cluster 4: older onset, DU, cardiac, lung, MSK, GI involvement, lcSSc, ATA > ACA	
	Cluster 5: male, younger onset, multi-organ involvements (cardiac, lung, GI, joint), dcSSc, ATA >ACA	
	Cluster 6: male, youngest onset, most aggressive, multi-organ involvement (cardiac, lung, renal, GI, MSK), dcSSc, ATA	
Intrinsic gene signature	Normal like	(27)
	Inflammatory	
	Fibroproliferative	
Monocyte subset	Cluster 1 (high CD16+ monocyte, low memory B cell subsets): lcSSc	(28)
	Cluster 2 (high classical monocytes): dcSSc, high mRSS	
	Cluster 3 (high memory B cells): often no skin involvement	
	Cluster 4 (low classical monocytes): often no skin involvement	
T-helper cells	Few immune abnormalities: gastrointestinal involvement, digital ulcer	(29)
	Treg-dominant group: anti-RNA polymerase III Ab, less digital ulcer and less gastrointestinal involvement	
	Tfh-dominant group: progressive skin sclerosis, gastrointestinal involvement, digital ulcer, late NFC pattern	
Gut microbiomes	**SSc cutanous subtypes and GI microbiome (species level)**	(30)
	LcSSc: ↓*Firmicutes prausnitzii*	
	DcSSc: ↑*Veillonella parvula, Klebsiella pneumoniae: dcSSc*	
	**SSc GI involvement and GI microbiome (genus level)**	(31)
	Milder GI symptoms: ↑*Lactobacillus, ↑ Clostridium*	
	More severe GI symptoms: ↑*Prevotella*	
	**SSc disease duration and GI microbiome (genus level)**	(32)
	Early SSc: *↑ Lactobacillus, ↑ Streptococcus, ↑ Blautia, ↓ Bacteroides, ↓ Sutterella*	
	Long-standing SSc: *↑ Lactobacillus, ↑ Streptococcus, ↓ Odoribacter, ↓ Sutterella*	
Proteomics	DcSSc with higher MRSS: upregulation of IGFBP-2, FSTL3, SPON1, ST2	(33)
	LcSSc with PAH: upregulation of FSTL3 and Midkine	(34)

*lc, limited cutaneous; dc, diffuse cutaneous; ACA, anti-centromere antibody; ATA, anti-topoisomerase antibody; ANA, anti-nuclear antibody; SSc, systemic sclerosis; Ab, antibody; ILD, interstitial lung disease; PAH, pulmonary arterial hypertension; PH, pulmonary hypertension; RP, Raynaud's phenomenon; SRC, scleroderma renal crisis; PM, polymyositis; DM, dermatomyositis; VEDOSS, very early diagnosis of SSc; NFC, nailfold capillaroscopy; MSK, musculoskeletal; GI, gastrointestinal; mRSS, modified Rodnan skin score; DU, digital ulcer*.

## SSc Molecular Stratification

### SSc Classification Based on Circulating Immune Cells

Van der Kroef et al. showed that prior to the onset of skin fibrosis and other organ manifestations, patients with Raynaud's phenomenon, positivity for SSc-specific autoantibodies and/ or specific NFC patterns, were shown to have different immune cell subset frequencies (Table 1) (28). Hierarchical cluster analysis showed that circulating immune cell population could be used to distinguish different SSc subsets into 4 different clusters, namely cluster 1 (high CD16+ monocytes and low memory B cells), cluster 2 (increased classical monocytes), cluster 3 (increased memory B cells), and cluster 4 (lower classical monocytes) (28). The different clusters were associated with different clinical features, for example limited cutaneous involvement in cluster 1 and no skin involvement in cluster 3 and 4. In contrast, cluster 2 was enriched in patients with ILD and diffuse cutaneous involvement (28). Future studies should further investigate the value of cellular phenotyping in relation to disease progression and treatment response.

### SSc Classification Based on Intrinsic Gene Subsets

SSc classification based on gene expression phenotyping were previously described, namely the fibroproliferative, inflammatory, normal-like intrinsic gene subsets (Table 1) (27, 35). Serial biopsies of skin specimen showed that the intrinsic gene subsets were inherent and stable features of the disease, suggesting distinct pathogenic processes between patients (27, 35). More recent studies by Skaug et al. showed that immune cell and fibroblast signatures changed over time in early dcSSc (within 3 years of disease onset) with a tendency toward normalization as the immune cell and fibroblast signatures declined at follow up (36). This could inform future clinical trials to stratify patients in early disease.

Within an individual with SSc, the intrinsic gene subsets were shown to be consistent across the different skin biopsy sites regardless of the clinical involvement (thickened skin or morphologically normal skin) (27, 35). In addition, the intrinsic gene subsets were demonstrated to be conserved across tissues such as the esophagus and skin (37). This highlights common pathogenic processes in SSc across different tissues. Nevertheless, the tissue microenvironment plays an important role in the immune-fibrotic axes (38). By using functional genomic network analysis, Taroni et al. identified a distinct lung specific innate immune process which suggests certain gene pairs are more likely to interact in a particular tissue than the others (38).

### Treatment Response in Relation to Intrinsic Gene Subsets

Molecular phenotyping of SSc patients has the potential to guide therapeutic approaches, specifically by selecting treatments that individual patients are most likely to respond based on their unique intrinsic gene subsets. Intrinsic gene subsets at baseline have been shown to potentially be predictive of therapeutic responses. Several studies have investigated the relationship between intrinsic gene subsets and specific treatment responses (Table 2).

**Table 2:** Stratification in relation to treatment responses.

**Medications**	**Improvers**	**Non-improvers**	**Study design^∧^**	**Tissue specimens**	**References**
Imatinib	Baseline high fibroproliferative related gene expression (phosphorylated PDGFRβ and Abl) that decreased post-treatment in improvers	N.A.	Longitudinal (*n*, 2)	Skin biopsy (lesional) at baseline and during therapy	(39)
Nilotinib	Baseline high expression of TGFβR and PDGFRβ signaling genes that decreased post-treatment in improvers	Baseline low expression of PDGFRβ signaling genes	Longitudinal (*n*, 6)	Skin biopsy (lesional) at baseline and during therapy	(40)
Dasatinib	Baseline normal like or fibroproliferative subset in improvers	Baseline inflammatory subset	Longitudinal (*n*, 12)	Skin biopsy (lesional and non-lesional) at baseline and during therapy	(41)
Fresolimumab	Baseline high TGFβ-regulated gene thrombospondin-1 expression that decreased post-treatment in improvers	Baseline high immune related genes	Longitudinal (*n*, 15)	Skin biopsy (lesional) at baseline and during therapy	(38), (42)
Mycophenolate mofetil	Baseline inflammatory subset in improvers	Baseline fibropliferative or normal like subset	Longitudinal (*n*, 9)	Skin biopsy (lesional and non-lesional) at baseline and during therapy	(43)
Abatacept	Baseline inflammatory subset with high levels of CD28 signaling in improvers	Baseline normal like subset with low levels of CD28 signaling	Longitudinal (*n*, 6)	Skin biopsy (lesional) at baseline and during therapy	(44)
Rituximab	N.A.	Variable subsets (inflammatory, fibroproliferative, normal like); no change in gene expression post-treatment	Longitudinal (*n*, 13)	Skin biopsy (lesional) at baseline and during therapy	(27)
Mycophenolate mofetil and cyclophosphamide	Baseline higher IFN-inducible protein score	Baseline lower IFN-inducible protein score	Longitudinal (*n*, 133)	Serum at baseline	(45)

∧ *Longitudinal study designs: tissue specimens were obtained serially at baseline and during therapies; n, sample size defined as number of patients with treatment and tissue specimens; N.A, not available*.

SSc patients in the fibroproliferative, but not the inflammatory gene subsets were shown to respond to tyrosine kinase inhibitors (TKI). TKI were explored as therapies for SSc because of the central role of tyrosine kinases in the pathogenesis of fibrosis (46). Imatinib is a small molecule TKI that antagonize c-Abl, a downstream mediator of PDGF and TGFβ receptors (R) (46). Use of Imatinib as experimental drug in SSc was previously reported in patients with dcSSc and clinical response to imatinib showed reduced expressions of genes typically found in the fibroproliferative subset (39). Similarly, responses to nilotinib, another TKI, were seen in patients with higher baseline expression of genes associated with TGFβR and PDGFR signaling, which significantly decreased in the improvers (*n* = 4, out of 6 patients) (40). In a more recent trial analyzing response to dasatinib, improvers (*n* = 3, out of total of 12 subjects) mapped to the fibroproliferative or normal-like subsets, whereas most of the non-improvers (*n* = 7, out of 9 non-improvers) were in the inflammatory subsets (41).

Janus kinase (JAK), which is a non-receptor tyrosine kinase that transduce cytokine signals via phosphorylation of STATs, has been suggested in pre-clinical studies to play a role in the pathogenesis of SSc through either pro-inflammatory or pro-fibrotic signals to the target cells (47). Gene expression profiling analysis confirmed elevated IL6/JAK/STAT and tofacitinib gene signatures in skin biopsies from the previously defined inflammatory subset of dcSSc patients, as compared to healthy controls (48). A pilot, single-center study of patients (*n* = 10, case series) evaluated the use tofacitinib, which inhibits primary JAK1/3 signaling in dcSSc with refractory skin thickness (49). The results demonstrated significant modified Rodnan skin score (mRSS) improvement in the first month suggesting its role as an effective immunosuppressant in refractory dcSSc with progressive skin thickness. PhaseI/II randomized controlled trial (NCT03274076) by Khanna et al. is ongoing, with initial results showing safety and a trend toward mRSS improvement (50). Further studies are needed to confirm the efficacy of tofacitinib and to evaluate its response in relation to inflammatory and fibrotic gene signatures.

In contrast to improvers to TKI, improvers to immunosuppressive medications were likely to be in the inflammatory gene subsets. Responders to mycophenolate mofetil (MMF) which targets lymphocyte proliferation (51), mapped to the inflammatory gene subset (*n* = 4, out of 7 improvers), whereas the non-improvers mapped to the fibroproliferative gene subset (n=2 subjects) (43). Likewise, responders to abatacept, which inhibits T cell activation by blocking CD80/CD86 interaction with CD28 (52), were in the inflammatory gene subset (*n* = 4 out of 5 improvers) and had higher baseline levels of CD28 signaling. The non-improver (*n* = 1) was in the normal-like gene subset with lower baseline levels of CD28 signaling (44).

Fresolimumab targets TGFβ signaling, with high baseline levels of TGFβ-regulated gene thrombospondin-1 (THBS1) that declined in patients with improved skin scores (42). Taroni et al. performed functional genomic meta-analysis, specifically functional genomic networks and machine learning of publicly available gene expression data from clinical trials of different therapeutics, including MMF and fresolimumab (53). While improvers to fresolimumab had high baseline TGFβ-related genes, non-improvers had elevated baseline levels of immune-related genes (53). Conversely, MMF improvers had high baseline immune-related genes that decreased post treatment (53). This study highlights the significance of genome-wide gene expression data gathered in clinical trials, which provides insight into the functional consequences of treatment and may be used to tailor treatment approaches.

## Multi-Omic Stratification of SSc: Toward Precision Medicine

The intrinsic complexity of SSc with heterogeneous manifestations necessitates a more strategic approach to thoroughly understand the underlying molecular mechanisms and to guide therapeutic approaches. Multiple-omics approaches from individual patients should be the direction of future work. Integration of high dimensional data encompassing information from transcriptomics, as well as genomics, epigenomics, proteomics, cytomics and microbiomics could lead to a more granular stratification system.

Through transcriptomic analysis, molecular signatures of SSc patients have been identified as described above. In addition, transcriptomic analysis has also revealed potential biomarkers with cross-sectional relationship with mRSS and may shed light into the disease pathogenesis (54). Two of the genes, cartilage oligomeric protein (COMP) and thrombospondin-1 (THBS1), are known to be regulated by transforming growth factor-β (TGFβ), whereas the other two genes, interferon-induced protein 44 (IFI44), and sialoadhesin (SIGLEC1) are known to be regulated by interferon (IFN) (54). More recently, systemic gene expression profiling through high throughput unsupervised clustering analysis has identified multiple genes as potential pharmacodynamic biomarkers in SSc skin (55). The identified genes were not limited to TGF? and IFN-regulated genes, but also MHC class I, proteasome, antigen processing, macrophage and vascular marker genes (55). These results highlight the roles of macrophage driven and vascular injury pathways in driving the disease process leading to fibrosis (55).

Technological advances in genomics, such as genome-wide association study (GWAS) and candidate gene approach (CGA) have highlighted important SSc susceptibility genes and non-HLA susceptibility genes (56). The majority of SSc susceptibility loci were found to be involved in innate or adaptive immune system (56). In addition, meta-analysis of GWAS revealed molecular pathways potentially involved in vasculopathy and fibrosis, both of which are central in the pathogenesis of SSc (57).

Although genetics play an important role in SSc pathogenesis, genetic factors alone are not sufficient to explain the disease occurrence, as there is low concordance rate of SSc among monozygotic twins (58). It is believed that environmental factors play an important role in the disease pathogenesis possibly through epigenetic regulation mediated through modifications in DNA, histone and non-coding RNAs (ncRNAs) (58, 59). However, the underlying pathophysiology linking genetic factors, epigenetic and environmental factors are still not fully understood.

The cellular responses to genetic, epigenetic and environment factors are reflected in the proteomic profiles. Accumulating data in high throughput proteomics have pointed to number of proteins and pathways associated with SSc progression and pathogenesis (60). Through progress in aptamer-based proteomic technology, a large array of serum protein was identified and could potentially be used as biomarkers in SSc to assess clinical progress as a number of differentially expressed proteins were found to correlate with mRSS (33). Differential expressions of proteins (midkine and FSTL3) were found in SSc patients with PAH and could potentially serve as a PAH biomarker and promising drug target (34). Type I interferons (IFNs), which are key regulators of innate immunity, play a role in the pathophysiology of SSc (61). Type I IFN signature was found in patients with very early SSc (before overt skin fibrosis), ATA and anti-U1 RNP antibodies (62, 63). High IFN-inducible chemokine levels were correlated with more severe skin, lung and muscle involvement in SSc (64). IFN-inducible proteins were demonstrated to have promising prognostic value in predicting treatment response (45, 61, 65).

Phase I trial of anifrolumab for SSc showed suppressed IFN signature in whole blood and skin, and this finding corresponded to suppression of T cell activation and collagen accumulation (66, 67). These shed light to the promising potential of using peripheral markers (e.g. high or low IFN signatures) to stratify patients for targeted treatment. More recently, serum proteins were shown to potentially be useful to guide therapeutic approaches in SSc-ILD patients (45). SSc-ILD patients with higher score of serum interferon-inducible proteins (IFNγ-inducible10-kd protein, monokine induced by IFNγ, monocyte chemotactic protein 2, β_2_-microglobulin, tumor necrosis factor receptor type II, and macrophage inflammatory protein 3β) responded better to immunosuppression (45). Future prospective longitudinal clinical studies are needed to evaluate the prognostic values of various candidate proteomic biomarkers in clinical practice.

In comparison to above techniques, cytomics allows simultaneous analysis of a number of parameters. It could be potentially used to shed light into the pathophysiology of SSc. High dimensional cytometry has proven to be a powerful tool to quantify large number of immune cell subsets and analyze their correlation with clinical markers (28, 68, 69). The frequency of monocyte subsets was found to be correlated with disease severity in SSc and changes in monocyte frequencies were already noted in the early phase of SSc disease in the pre-fibrotic stage. (28) Our group investigated blood mononuclear cells from SSc patients using mass cytometry and transcriptomic analysis (68). Unsupervised clustering analysis were performed to identify nodes composed of similar cells, and the results revealed significant differences in the frequencies of T and B cell subsets in SSc subsets, as well as compared to healthy controls (68). In patients with ILD compared to those without ILD, we found increased nodes representing CD4+ T cells expressing CCR4 and ICOS, but decreased nodes representing mucosal associated invariant T cells (68). In addition, based on peripheral blood immune cell phenotypes and organ involvement, Kubo S et al. stratified SSc patients into 3 groups: Treg-dominant group, Tfh-dominant group and fewer abnormalities group (Table 1) (29). Future studies could evaluate the potential role of immune cell phenotypes to prognosticate therapeutic response, e.g., role of targeted therapy for B cells by rituximab in Tfh-plasmablast dominant group. Despite these advances, identification of high dimensional biomarkers to clearly stratify patients with SSc remains an unmet need. The Extended Polydimensional Immunome Characterization (EPIC), a web-based discovery tool could be deployed for comprehensive analyses of single cell dataset to identify high dimensional biomarkers in SSc patients in comparison to healthy datasets (69).

Dysbiosis of the GI microbiome is known to have systemic effect on the immune system in SSc (70). Two culture-independent metagenomic sequencing technologies have been used to characterize the GI microbiome. Most commonly reported technology is the 16S RNA sequencing, which enables bacterial identification (answers the question “who are they?”). Whole-genome shotgun sequencing enables the identification of the gene and their metabolic and enzymatic pathways (answers the question “what are they doing?”). In SSc compared to healthy controls, consistent observations using both approaches have shown reduced abundance of *Bacteroides* species (which protects host from mucosal inflammation), *Clostridium* and *Faecalibacterium* species (butyrate-producing organisms that enhance epithelial barrier function), and increased abundance of *Lactobacillus* (implicated in SSc GI dysmotility) and *Bifidobacterium* species (30, 70). Alpha diversity, which is the complexity of microbiome composition within individuals of a group, was suggested to be decreased with SSc patients with more severe disease, longer disease duration and dcSSc (Table 1) (30–32, 71–73). There have been a few small clinical trials on GI-microbiome therapeutic interventions in SSc (74–76). In a placebo-controlled trial of probiotics, Low et al. found that baseline microbiome composition and probiotics were independently associated with GI symptom improvement. (75) These suggest a potential role of GI microbiome modulation to improve GI symptoms. Longitudinal studies of GI microbiome in SSc are needed to understand the contribution of microbiome alteration to the development of GI and extra-intestinal manifestations of SSc.

With the tremendous amount of multi-omics data in SSc, advances in machine learning has made it possible to integrate high dimensional data with cutting-edge computational tools. Systems biology based approach has the potential to condense multiple-omics data to derive meaningful molecular interaction network and facilitate better capture of SSc complex pathogenesis (77). Although it is challenging to delineate the modular organization at the molecular level, multiple integration strategies have been developed to analyze regulatory relationships between each omic layer (78). Future integration of multi-omics data may improve our understanding of complex SSc pathogenesis and distinguish distinct patient subtypes.

## Conclusion and Unmet Need

SSc is a complex multisystemic disease with heterogenous clinical manifestations and characteristics. Advances in transcriptomics have led to identification of distinct SSc molecular gene signatures. Tremendous amount of multi-omics data has emerged in SSc field and machine learning could be deployed to integrate and analyze multi-omic data in SSc to develop a more granular classification system.

Challenges in multi-omic data analysis include the rarity of SSc disease and hence its limited sample size. Furthermore, lack of clinical information, e.g., disease duration or treatment history, further hinders clinical phenotyping of the subjects in publicly available datasets. In the future, a more comprehensive longitudinal study of SSc patients and collaborative effort to integrate high dimensional data would be pivotal to create an SSc atlas at global level to gain more insights into disease etiology, prognosis and progression. This may help the characterization of heterogenous SSc patients and personalization of therapeutic approaches toward precision medicine.

## Author Contributions

AL and SA contributed to conception and design of the study. MN wrote the first draft of the manuscript. VC contributed to manuscript revision and editing. All authors contributed to manuscript revision, read, and approved the submitted version.

## Funding

MN holds Seah Cheng Siang Memorial Research Award; AL holds a National Medical Research Council (NMRC) Clinical Scientist Award (grant number: MOH-CSAINV19may-0005); SA holds grant support from NMRC (NMRC/OFLCG/002/2018, CIRG19may-0052, MOH-STaR19nov-0002, COVID19TUG21-0120, NMRC/CG1/006/2021-KKH/MOH-000988-00), and A*STAR (H22P0M0003).

## Conflict of Interest

The authors declare that the research was conducted in the absence of any commercial or financial relationships that could be construed as a potential conflict of interest.

## Publisher's Note

All claims expressed in this article are solely those of the authors and do not necessarily represent those of their affiliated organizations, or those of the publisher, the editors and the reviewers. Any product that may be evaluated in this article, or claim that may be made by its manufacturer, is not guaranteed or endorsed by the publisher.
